# IgE-mediated enhancement of CD4^+^ T cell responses requires antigen presentation by CD8α^−^ conventional dendritic cells

**DOI:** 10.1038/srep28290

**Published:** 2016-06-16

**Authors:** Zhoujie Ding, Joakim S. Dahlin, Hui Xu, Birgitta Heyman

**Affiliations:** 1 Department of Medical Biochemistry and Microbiology, Uppsala University, Uppsala, Sweden

## Abstract

IgE, forming an immune complex with small proteins, can enhance the specific antibody and CD4^+^ T cell responses *in vivo*. The effects require the presence of CD23 (Fcε-receptor II)^+^ B cells, which capture IgE-complexed antigens (Ag) in the circulation and transport them to splenic B cell follicles. In addition, also CD11c^+^ cells, which do not express CD23, are required for IgE-mediated enhancement of T cell responses. This suggests that some type of dendritic cell obtains IgE-Ag complexes from B cells and presents antigenic peptides to T cells. To elucidate the nature of this dendritic cell, mice were immunized with ovalbumin (OVA)-specific IgE and OVA, and different populations of CD11c^+^ cells, obtained from the spleens four hours after immunization, were tested for their ability to present OVA. CD8α^−^ conventional dendritic cells (cDCs) were much more efficient in inducing specific CD4^+^ T cell proliferation *ex vivo* than were CD8α^+^ cDCs or plasmacytoid dendritic cells. Thus, IgE-Ag complexes administered intravenously are rapidly transported to the spleen by recirculating B cells where they are delivered to CD8α^−^ cDCs which induce proliferation of CD4^+^ T cells.

Antibodies (Ab) passively administered together with their specific antigen (Ag) can enhance or suppress the immune response against this Ag. This phenomenon is known as Ab-mediated feedback regulation^1^. Whether the responses will be up- or down-regulated is determined by the type of Ag and the Ab class. IgE is an example of an isotype which enhances Ab responses against small soluble Ag such as ovalbumin (OVA), bovine serum albumin (BSA), tetanus toxoid and diphtheria toxoid^2^,^3^,^4^,^5^,^6^,^7^,^8^. In addition, IgE can also enhance CD4^+^ T cell response against OVA^5^,^6^,^7^. These processes are dependent on the low affinity receptor for IgE, CD23^2^,^3^,^4^, and in order for IgE to be able to enhance Ab and T cell responses, CD23 must be expressed on B cells^5^,^6^. *In vitro*, both human and mouse B cells are able to process IgE-Ag complexes, acquired via CD23, and to present the Ag to CD4^+^ T cells^9^,^10^,^11^,^12^,^13^. Therefore, it was initially hypothesized that this mechanism explained also IgE-mediated enhancement of T cell proliferation *in vivo*. However, the observation that CD23^+^ B cells capture IgE-Ag complexes in the blood and transport them into splenic follicles, where IgE-Ag can be detected on follicular B cells after 30 min, offered an alternative explanation to the requirement for CD23^+^ B cells^6^,^7^, indicating that they transported rather than presented IgE-Ag. When this was tested experimentally, several lines of evidence suggested that CD11c^+^ cells and not CD23^+^ B cells presented IgE-complexed Ag to CD4^+^ T cells *in vivo*^7^: (i) IgE-Ag complexes did not induce proliferation of CD4^+^ T cells in mice where CD11c^+^ cells were depleted; (ii) spleen cells from mice immunized with IgE-Ag complexes *in vivo* could only stimulate T cell proliferation *ex vivo* if they contained CD11c^+^ cells while depletion of B cells did not abolish the Ag-presenting capacity; (iii) T cell proliferation in CD23^−/−^ mice, immunized with IgE-Ag, could be rescued by transfer of MHC-II-incompatible CD23^+^ B cells which would be able to transport, but not to present, antigenic peptides to T cells in the recipient mice.

There are three major subsets of CD11c^+^ cells in the mouse spleen: CD8α^−^ conventional dendritic cells (cDCs), CD8α^+^ cDCs, and plasmacytoid dendritic cells (pDCs)^14^,^15^,^16^. CD8α^−^ cDCs and CD8α^+^ cDCs express high levels of CD11c while pDCs express intermediate levels. CD8α^−^ cDCs are located in the marginal zone bridging channels^17^ and migrate to the T cell zone after administration of lipopolysaccharide, Toxoplasma gondii or high doses of sheep red blood cells^18^,^19^,^20^,^21^. CD8α^+^ cDCs are less abundant than CD8α^−^ cDCs and constitute about 30% of CD11c^high^ cells. They are found in the marginal zone, the T cell zone, and the red pulp^14^,^22^,^23^. pDCs are not considered professional antigen presenting cells (APCs) but can prime CD4^+^ T cells or cross-prime CD8^+^ T cells under certain conditions^24^,^25^,^26^. They are more well-known for producing high levels of type I interferon after viral infections^25^,^27^,^28^.

Here, we have investigated which subset of CD11c^+^ cells is able to present Ag to CD4^+^ T cells in mice immunized with IgE-Ag complexes. The results show that CD8α^−^ cDCs are the most important APCs in this situation.

## Results

### IgE anti-OVA enhances specific IgG and CD4^+^ T cell responses

**Figure 1:**
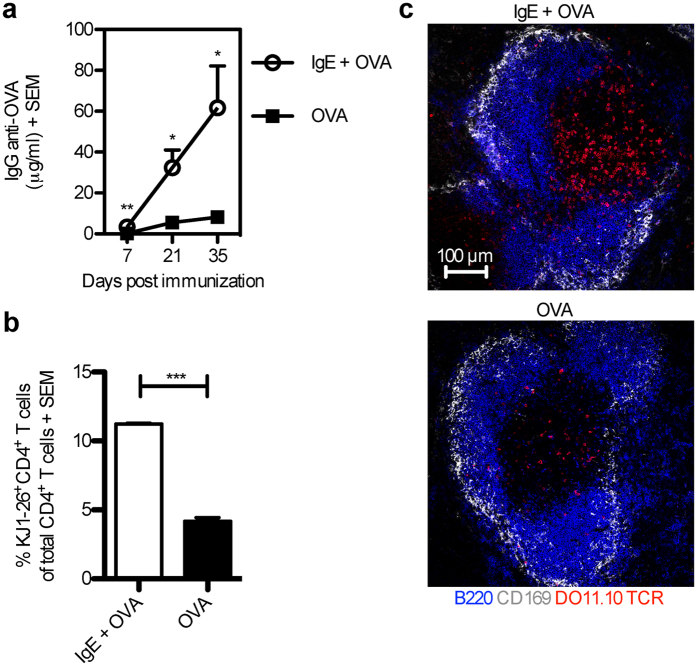
IgE anti-OVA enhances both OVA-specific IgG and CD4^+^ T cell responses. (**a**) BALB/c mice were immunized with 50 μg IgE anti-OVA pre-mixed with 20 μg OVA (n = 7), or 20 μg OVA alone (n = 7). Sera from d 7, 21, and 35 after immunization were analysed for IgG anti-OVA by ELISA. (**b**) BALB/c mice were adoptively transferred with splenocytes from DO11.10 mice one day before administration of 50 μg IgE anti-OVA pre-mixed with 20 μg OVA (n = 3) or 20 μg OVA alone (n = 3). Spleens were harvested 3 days after immunization and half of each spleen was analysed for proliferation of OVA-specific CD4^+^ T cells by flow cytometry. The gating strategy is shown in Supplementary Fig. S2. Percentages of KJ1-26^+^CD4^+^ T cells among total CD4^+^ T cells of each group were then quantified. (**c**) The other half of each spleen as in (**b**) was frozen and spleen sections were stained and analysed by confocal microscopy. B220, blue; CD169, grey; DO11.10 TCR, red. Images show T cell areas (640 μm × 640 μm) representative of 6 T cell zones from 2 non-consecutive sections per sample in each group. Scale bar represents 100 μm. (**a,b**) Data are representative of three independent experiments and are shown as mean + SEM. Significance was determined between the group immunized with IgE-OVA complexes and the group immunized with OVA alone by Student’s *t*-test. *p < 0.05; **p < 0.01; ***p < 0.001.

Previous studies have used 2,4,6-trinitrophenol (TNP)-conjugated Ag together with monoclonal IgE anti-TNP to study IgE-mediated enhancement of immune responses^2^,^3^,^4^,^5^,^6^,^7^,^29^,^30^. Here, we used a system in which immune complexes were formed between monoclonal IgE anti-OVA and OVA. BALB/c mice were immunized with OVA alone or OVA pre-mixed with IgE anti-OVA and the Ab and T cell responses were analysed. Similarly to IgE anti-TNP, IgE anti-OVA enhanced the OVA-specific IgG- and CD4^+^ T cell-responses (Fig. 1). As expected from previous studies^5^,^7^, no IgE-mediated enhancement of T cell proliferation was seen in CD23^−/−^ mice (Supplementary Fig. S1).

### Ag administered together with specific IgE is detected in splenic B cell follicles after 0.5 h and persists for at least 4 h

**Figure 2:**
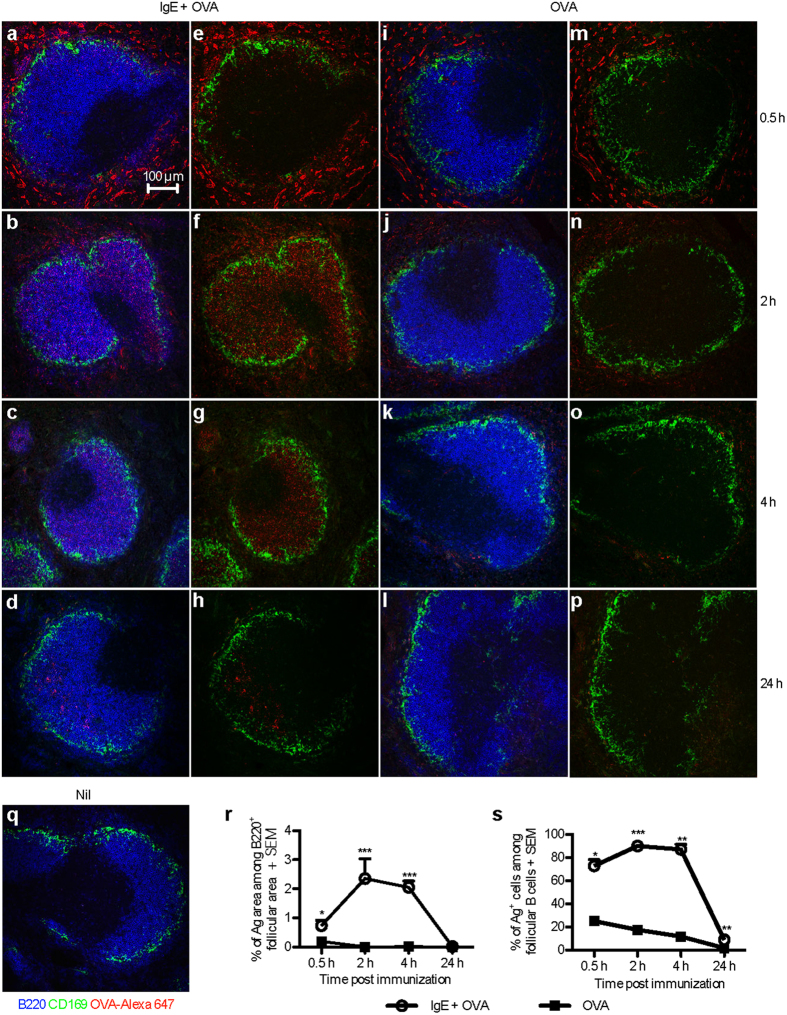
OVA administered together with specific IgE is rapidly transported to splenic B cell follicles. BALB/c mice were immunized with 50 μg IgE anti-OVA pre-mixed with 150 μg OVA-Alexa 647 (n = 2 per time point) (**a–h**), 150 μg OVA-Alexa 647 alone (n = 2 per time point) (**i–p**), or left unimmunized (**q**). Spleens were harvested 0.5, 2, 4, or 24 h after immunization. Non-consecutive sections of spleens were stained and analysed by confocal microscopy. B220^+^ B cells, blue; CD169^+^ metallophilic macrophages, green; OVA-Alexa 647, red. (**a–d**,**i–l,q**) All colors are shown. (**e–h,m–p**) All colors expect blue are shown. (**a–q**) Images show follicular areas (640 μm × 640 μm) representative of 3–4 follicular areas from 2 non-consecutive sections per sample in each group. Scale bar represents 100 μm. Data represent one experiment at 0.5 h and 2 h and two experiments at 4 h and 24 h. (**r**) Quantification of the Ag^+^ area within the B220^+^ follicular area. (**s**) Percentages of Ag^+^ cells among follicular B cells was analysed by flow cytometry on splenocytes from the other half of each spleen in (**a–p**). Follicular B cells are gated as B220^+^CD21^+^CD23^high^ cells (Supplementary Fig. S3). (**r,s**) Data are shown as mean + SEM. Significance was determined between the group immunized with IgE-OVA complexes and the group immunized with OVA alone by Student’s *t*-test. *p < 0.05; ***p < 0.001.

Mice were immunized with Alexa Fluor 647-conjugated OVA (OVA-Alexa 647; an Ag which can be detected intracellularly) with or without IgE anti-OVA and localization of Ag in their spleens was analysed. Ag was found in the B cell follicles of mice immunized with IgE-OVA-Alexa 647 complexes after 0.5 h (Fig. 2a,e), and the amount of Ag in the follicles increased after 2 h (Fig. 2b,f) and 4 h (Fig. 2c,g) and diminished after 24 h (Fig. 2d,h). Very little Ag was detected inside the B cell follicles of mice immunized with OVA-Alexa 647 alone (Fig. 2i–p). As expected, no OVA signal was detected in unimmunized mice (Fig. 2q). Quantification of the Ag positive areas within the B220^+^ follicles confirmed that Ag localization inside the follicles was enhanced by IgE anti-OVA 0.5–4 h after immunization (Fig. 2r). Moreover, at these time points around 70% of the follicular B cells from mice immunized with IgE-OVA-Alexa 647 complexes were positive for Ag (Fig. 2s). To conclude, IgE anti-OVA facilitates the transport of OVA into B cell follicles, and OVA is found in the B cell follicles at least until 4 h after immunization.

### CD8α^−^ cDCs are the cells primarily presenting IgE-complexed Ag to CD4^+^ T cells

**Figure 3:**
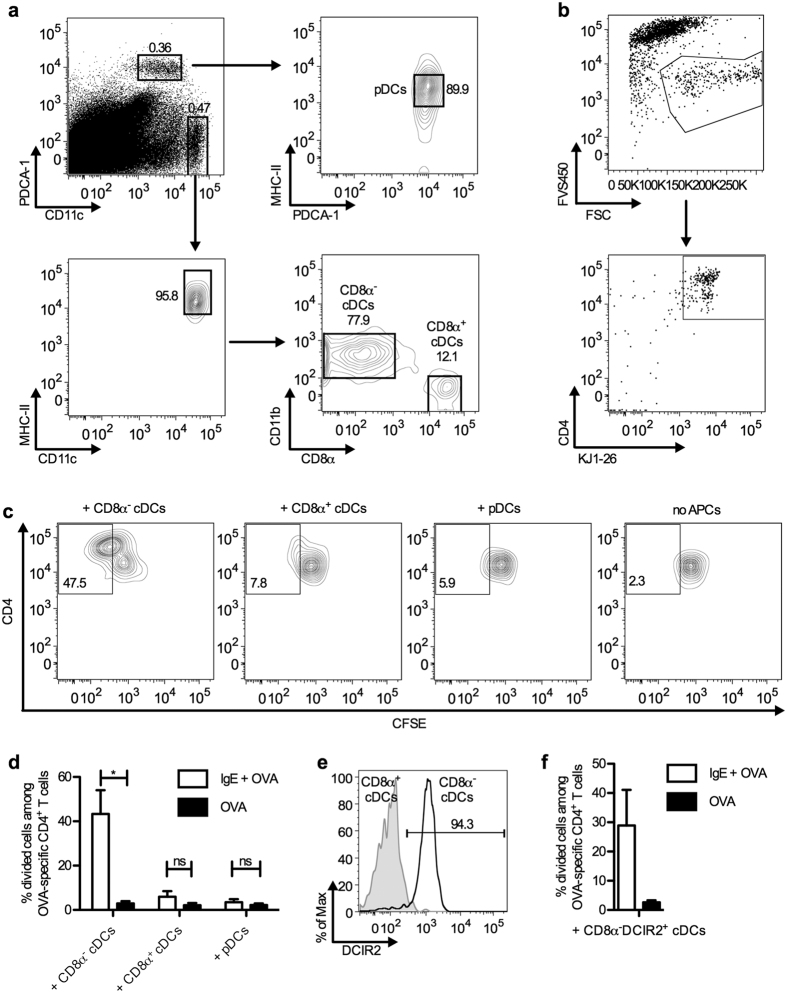
CD8α^−^ cDCs/CD8α^−^DCIR2^+^ cDCs are the cells primarily presenting IgE-complexed Ag to specific CD4^+^ T cells. BALB/c mice were immunized with 250 μg IgE anti-OVA pre-mixed with 100 μg OVA, or with 100 μg OVA. Four hours after immunization, their spleens were harvested and the splenocytes were sorted into different APC populations: (**a**) CD8α^−^ cDCs (PDCA-1^−^CD11c^high^MHC-II^high^CD11b^+^CD8α^−^), CD8α^+^ cDCs (PDCA-1^−^CD11c^high^MHC-II^high^CD11b^−^CD8α^+^) and pDCs (PDCA-1^+^CD11c^int^MHC-II^int^), or (**e**) CD8α^−^DCIR2^+^ cDCs (first gated for CD8α^−^ cDCs as above and then on DCIR2). Each of these APC populations was co-cultured with 5000 CFSE-labeled CD4^+^ T cells from DO11.10 spleens. After incubation at 37 °C for 3 days, the number of proliferating T cells was determined by flow cytometry measuring CFSE dilution (divided cells) among OVA-specific CD4^+^ T cells. (**b**) Gating of OVA-specific CD4^+^ T cells. Dead cells were excluded by FVS450 staining. OVA-specific CD4^+^ T cells were gated as CD4^+^KJ1-26^+^ among live cells. (**c**) Gating of divided cells among OVA-specific CD4^+^ T cells. Numbers indicate percentages of divided cells among OVA-specific CD4^+^ T cells. CD4^+^ T cells cultured without APC were used as negative controls. (**d,f**) Percentages of divided cells among OVA-specific CD4^+^ T cells, incubated with the indicated APCs. Data are pooled from three independent experiments using 5000 or 1200 CD8α^−^ cDCs, CD8α^+^ cDCs, or pDCs as APCs (**d**) and from two independent experiments using 5000 CD8α^−^DCIR2^+^ cDCs (**f**). Data are shown as mean + SEM. Significance was determined between the groups immunized with IgE-OVA complexes and OVA alone by Student’s *t*-test. *p < 0.05; no significance (ns), p > 0.05.

The inability of B cells from mice immunized with IgE and Ag to present Ag to T cells has been demonstrated previously^7^. Mice were immunized with monoclonal IgE anti-TNP + OVA-TNP and spleen cells were removed after 4 h and depleted of CD19^+^ cells (B cells) or CD11c^+^ cells. When tested for ability to induce proliferation in DO11.10 cells in a system analogous to the one used in the present report, spleen cells depleted of B cells retained the same antigen presenting capacity as total spleen cells whereas spleen cells depleted of CD11c^+^ cells completely lost their antigen presenting capacity^7^. To elucidate which subset of CD11c^+^ cells was involved, we immunized BALB/c mice with IgE-OVA complexes or OVA alone, harvested spleens 4 h later and tested the various APC populations for ability to induce proliferation of OVA-specific T cells *ex vivo*. Splenocytes were sorted into CD8α^−^ cDCs, CD8α^+^ cDCs, and pDCs (Fig. 3a). The three subtypes of CD11c^+^ cells were co-cultured with CFSE-labeled CD4^+^ T cells from DO11.10 mice carrying an OVA-specific TCR. No Ag or ligand was added to the cell cultures during incubation and therefore OVA-peptides presented on MHC-II on the APCs *in vitro* were derived from Ag taken up by the cells *in vivo*. Three days after initiation of the cultures, OVA-specific CD4^+^ T cells were gated and the percentages of divided cells among the total OVA-specific CD4^+^ T cells were quantified as exemplified in Fig. 3b,c. Clearly, CD8α^−^ cDCs were by far the most efficient inducers of T cell proliferation (Fig. 3d). Dendritic cell inhibitory receptor 2 (DCIR2) is a specific surface marker for CD8α^−^ cDCs in mouse and can be recognized by the 33D1 monoclonal Ab (mAb)^31^. More than 94% of the CD8α^−^ cDCs were positive for DCIR2 whereas CD8α^+^ cDCs expressed little DCIR2 (Fig. 3e), confirming that this marker was indeed specific for CD8α^−^ cDCs. As an alternative approach, CD8α^−^DCIR2^+^ cDCs from BALB/c mice, immunized as above, were sorted and used as APCs. CD8α^−^DCIR2^+^ cDCs isolated from mice immunized with IgE-OVA complexes induced a higher T cell proliferation than those from mice immunized with OVA alone (Fig. 3f).

Taken together, CD8α^−^ cDCs obtained from mice immunized with IgE-OVA, but not OVA alone, induced proliferation of OVA-specific CD4^+^ T cells (Fig. 3d,f). In contrast, neither CD8α^+^ cDCs nor pDCs, taken from the same mice, induced significant T cell proliferation (Fig. 3d).

### CD8α^−^ cDCs do not express CD23

**Figure 4:**
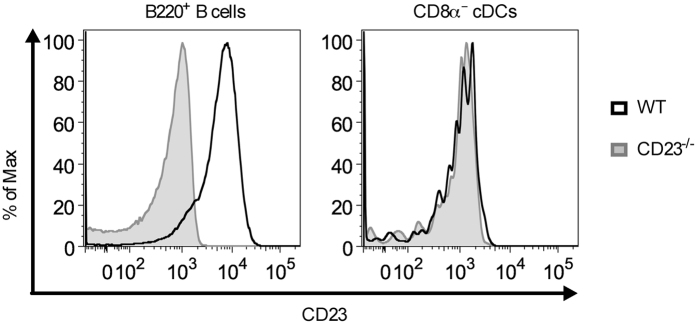
CD8α^−^ cDCs do not express CD23. Splenocytes from naïve wildtype (WT) BALB/c or CD23^−/−^ mice were stained and analysed by flow cytometry. B220^+^ B cells (B220^+^CD11c^−^) were gated as in Supplementary Fig. S4. CD8α^−^ cDCs were gated as in Fig. 3a. The expression of CD23 on B220^+^ B cells (left panel) and CD8α^−^ cDCs (PDCA-1^−^CD11c^high^MHC-II^high^CD11b^+^CD8α^−^; right panel) from the same wildtype or CD23^−/−^ mouse are shown as histograms. Data are representative of two independent experiments with 2–3 mice of each strain in each experiment.

In mice, only B cells and follicular dendritic cells have been reported to express CD23a^32^,^33^, which is the isoform required for IgE-mediated enhancement of Ab responses^29^. To exclude that CD8α^−^ cDC express CD23 and that their activation of CD4^+^ T cells was therefore due to direct capture and internalization of IgE-OVA complexes by ‘self’ CD23, we examined the expression of this receptor. B220^+^ B cells from wild-type mice, which served as positive controls, expressed high levels of CD23 whereas the levels of CD23 on CD8α^−^ cDCs were equally low in wild-type and CD23^−/−^ mice (Fig. 4). Therefore, CD8α^−^ cDCs must acquire IgE-Ag complexes from CD23^+^ B cells via other pathways than through direct capture by ‘self’ CD23.

### CD8α^−^ cDCs migrate from the marginal zone bridging channel into the T cell zone after immunization both with IgE-complexed OVA and uncomplexed OVA

**Figure 5:**
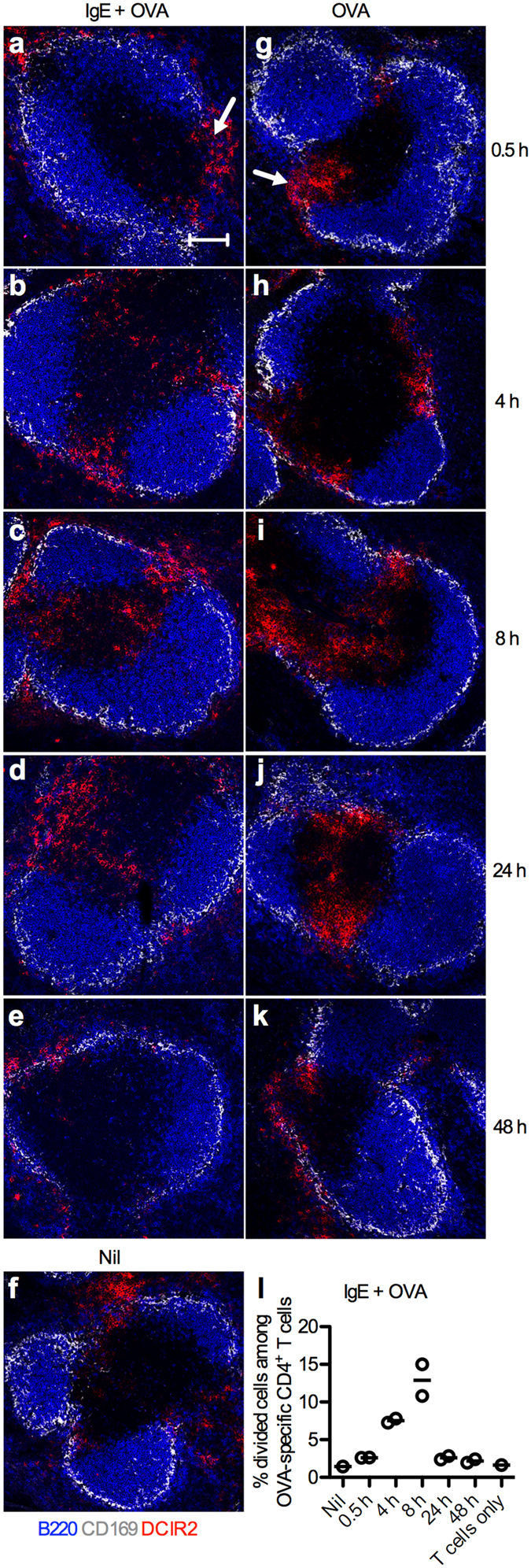
DCIR2^+^ cDCs migrate from the marginal zone bridging channel to the T cell zone after immunization. Spleens from BALB/c mice (n = 2 per time point) immunized with 250 μg IgE anti-OVA pre-mixed with 100 μg OVA or with 100 μg OVA alone were harvested after 0.5, 4, 8, 24, or 48 h. One unimmunized mouse (Nil) was used as control. (**a–k**) Half of each spleen was snap-frozen and non-consecutive spleen sections were stained and analyzed by confocal microscopy. Localization of DCIR2^+^ cDCs in spleens harvested at indicated time points after immunization was followed. B220^+^ B cells, blue; CD169^+^ metallophilic macrophages, grey; DCIR2^+^ cDCs, red. Marginal zone bridging channels are indicated with arrows in (**a,g**). Images show representative areas (640 μm × 640 μm) of 3–4 T cell zones from 2 non-consecutive sections of each sample in every group. Scale bar represents 100 μm. Data represent one experiment where mice were immunized with IgE-OVA or OVA alone and one where they were immunized with IgE-OVA. (**l**) The other halves of the spleens from mice immunized with IgE-OVA complexes in (**a–e**) were prepared into single cell suspensions and 6 × 10^5^ cells were used as APCs in co-cultures with 10^5^ CFSE-labeled CD4^+^ T cells isolated from DO11.10 splenocytes. Percentages of divided cells among OVA-specific CD4^+^ T cells after incubation for 3 days with APCs taken from an unimmunized mouse (Nil) or from mice immunized with IgE-OVA complexes are quantified by flow cytometry as shown in Fig. 3. CD4^+^ T cells cultured alone were used as negative control. Each circle represents one mouse and the lines represent the mean values.

The finding that CD8α^−^DCIR2^+^ cDCs overlapped with the CD8α^−^ cDC population, and that they were also efficient presenters of IgE-OVA (Fig. 3e,f) opened the possibility to stain spleen sections for these cells using the 33D1 mAb. The marginal zone bridging channel is a connection that penetrates the marginal zone envelope from the white pulp into the red pulp in the spleen^34^. To analyze migration of CD8α^−^ cDCs in our system, spleen sections from mice immunized with OVA, IgE-OVA complexes, or left unimmunized were stained for CD8α^−^ cDCs. In unimmunized mice, CD8α^−^ cDCs as expected resided in the marginal zone bridging channels (Fig. 5f). In immunized mice, no migration was obvious 0.5 h after immunization (Fig. 5a,g). However, 4 h after immunization, the CD8α^−^ cDCs started to migrate from the marginal zone bridging channels to the T cell zone (unstained area surrounded by the B cell follicles) (Fig. 5b) and localization of CD8α^−^ cDCs within the T cell zone was most pronounced after 8–24 h (Fig. 5c,d,i,j). After 48 h, CD8α^−^ cDCs could no longer be detected in the T cell zone (Fig. 5e,k). Interestingly, the migration patterns of CD8α^−^ cDCs in mice given IgE-OVA or OVA without IgE followed a similar pattern (Fig. 5g–k). In parallel to the analysis by confocal microscopy, splenocytes from the other half of the spleen of mice immunized with IgE-OVA complexes were used as APCs in a T cell proliferation assay. APCs obtained 8 h after immunization induced the highest percentage of cell division amongst OVA-specific CD4^+^ T cells (Fig. 5l). The temporal link between the migration of CD8α^−^ cDCs into the T cell zone and their ability to activate CD4^+^ T cells *ex vivo* supports the hypothesis that these cells present IgE-complexed Ag to CD4^+^ T cells in the T cell zone *in vivo*.

**Figure 6:**
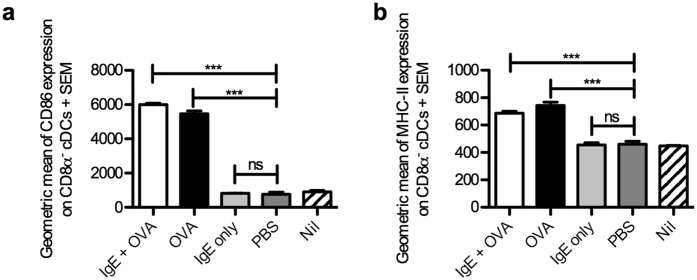
CD86 and MHC-II expression on CD8α^−^ cDCs 8 h after immunization. BALB/c mice were immunized with 250 μg IgE anti-OVA pre-mixed with 100 μg OVA (n = 3), 100 μg OVA alone (n = 3), 250 μg IgE anti-OVA alone (n = 3) or with PBS (n = 3). Mice left unimmunized (Nil) were used as negative control (n = 3). Spleens were harvested 8 h after immunization and prepared for flow cytometry analysis. CD8α^−^ cDCs were gated as in Fig. 3a (except that MHC-II was not used in the gating strategy when MHC-II expression was quantified). Geometric means of CD86 (**a**) or MHC-II (**b**) expression on CD8α^−^ cDCs from all groups were quantified. Data are representative of two independent experiments (except for the IgE alone-group which was only analysed in the experiment shown) and are shown as mean + SEM. Significance was determined between the groups immunized with IgE-OVA complexes, OVA alone or IgE and PBS by Student’s *t*-test. ***p < 0.01; no significance (ns), p > 0.05.

To test whether CD8α^−^ cDCs from mice immunized with OVA or IgE-OVA exhibited signs of activation *in vivo*, thus making them competent to activate CD4^+^ T cells, the expression of CD86 and MHC-II was analyzed 8 h after immunization. Both OVA and IgE-OVA induced upregulation of CD86 and MHC-II, whereas PBS or IgE did not increase the levels above those seen in unimmunized mice (Fig. 6).

## Discussion

This study has two central observations. First, it extends and confirms the finding that recirculating B cells rapidly transport IgE-complexed Ag to splenic follicles where the amount of Ag peaks 2–4 h after immunization. Second, it demonstrates that CD8α^−^ cDCs, but not CD8α^+^ cDCs or pDCs, obtained from mouse spleens 4–8 h after immunization with IgE-OVA (but not with OVA alone) induce proliferation of OVA-specific CD4^+^ T cells *ex vivo*. For blood-borne pathogens, as well as for Ag administered intravenously, the spleen is the most important secondary lymphoid organ. However, whereas localization of Ag to lymph node follicles has been well characterized^35^, it is less well understood how Ag is transported into splenic follicles. Small Ag can be distributed to follicles through conduits^36^, marginal zone B cells capture complement-coated Ag in the marginal zone via their complement receptors and deliver it to follicular dendritic cells^37^,^38^,^39^, and cognate B cells have been shown to capture intranasally administered virus-like particles and transport them to the spleen^40^. In addition to these processes, we have previously reported a novel role for CD23^+^ peripheral B cells. Already 5 minutes after immunization, these cells have bound IgE-Ag complexes^6^,^37^ and after 30 minutes Ag is found on follicular B cells in the splenic follicles^6^. This remarkable transportation capacity of peripheral B cells has here been studied in more detail, showing that both the amount of Ag in follicles and the percentage of Ag^+^ follicular B cells peak 2–4 h after immunization. IgE anti-TNP enhances responses to proteins over a wide range of epitope numbers (1–27 TNP/protein molecule)^2^,^7^,^8^,^41^,^42^. This suggests that the complex size is not critical and IgE anti-TNP enhances Ab and CD4^+^ T cell responses to OVA-TNP with 1–3 epitopes/OVA^7^,^8^,^41^ thus forming immune complexes that would be of similar sizes as those formed after administration of monoclonal IgE anti-OVA and OVA. The insensitivity to complex size of IgE-mediated enhancement should make the system flexible and also extremely potent since one IgE molecule/Ag molecule suffices for binding to CD23 on B cells and for subsequent transport into follicles.

Based on the kinetics of the Ag concentration in the follicles and the kinetics of spleen cells to present OVA to specific CD4^+^ T cells, spleens were harvested 4 h after immunization, separated into different dendritic cell populations and tested for ability to present Ag to OVA-specific CD4^+^ T cells *in vitro*. No Ag was added to the cell cultures and therefore OVA-peptides in the MHC-II molecules were derived from Ag taken up *in vivo*. In spite of this and of the low APC:CD4^+^ T cell ratio in the cultures (≤1:1), a 5–30-fold increase in T cell proliferation was observed when CD8α^−^ cDCs were added to the cultures. In contrast, no T cell proliferation was induced by CD8α^+^ cDCs or pDCs. These results are consistent with previous observations that CD8α^−^ cDCs preferentially present Ag to CD4^+^ T cells rather than cross-presenting it to CD8^+^ T cells^31^,^43^, a finding possibly explained by different intrinsic properties in the Ag-processing pathways of CD8α^−^ cDCs and CD8α^+^ cDCs^31^. CD8α^−^ cDCs express genes involved in MHC-II Ag presentation (e.g. cathepsins C, H, and Z, asparagine endopeptidase, and H2-Mbeta 1) whereas CD8α^+^ cDCs express genes related to MHC-I Ag presentation (e.g. Tap1, Tap2, and calreticulin), and after immunization, CD8α^−^ cDCs have much higher levels of MHC-II-peptide complexes on their surface than CD8α^+^ cDCs^31^.

CD8α^−^ cDCs from mice immunized with IgE-OVA or with OVA, migrate equally well into the T cell zone (Fig. 5) and also upregulate CD86 and MHC-II to the same degree (Fig. 6). Most likely the activation and migration are dependent on innate signals, possibly delivered via endotoxins in the OVA preparations^44^. This would be consistent with reports showing that cDCs migrate to the T cell zones after stimulation with TLR ligands^18^,^21^. In spite of the presence of CD8α^−^ cDCs in the T cell zones both in mice immunized with IgE-OVA or with OVA alone, only CD8α^−^ cDCs from IgE-OVA immunized mice are able to activate specific T cells (Figs 1b,c and 3d,f, Supplementary Fig. S1). This seems logical, since substantial amounts of Ag can only be found in the follicles of mice given IgE-OVA and presence of Ag would be a prerequisite for uptake and presentation of OVA peptides to CD4^+^ T cells. Thus, these observations emphasize the crucial role of the IgE/CD23-dependent transport of Ag from the blood to the follicles in generating the enhanced T-cell proliferation.

The details of delivery of IgE-OVA from the CD23^+^ B cells to the CD8α^−^ cDCs are not understood. Nevertheless, it is evident that Ag delivery does indeed take place since CD8α^−^ cDCs are able to present OVA peptides to T cells within 4 h of immunization with IgE-OVA (Fig. 3). We have previously shown that B cells transferred from IgE-anti-TNP + OVA-TNP-immunized mice can rescue DO11.10 T cell proliferation in CD23^−/−^ mice^7^. Moreover, proving the point that these B cells did not present (but only transport) the Ag, MHC-II incompatible B cells transferred to CD23^−/−^ mice could rescue DO11.10 T cell proliferation^7^. The delivery of IgE-Ag could take place in the marginal zone bridging channels or at the T-B border. Clearly, CD23^+^ B cells must be involved in this chain of events because no enhancement of CD4^+^ T cell proliferation or Ab responses takes place in their absence^5^,^6^. The majority of follicular B cells from mice immunized with IgE-OVA, but not with OVA alone, are Ag^+^ 0.5–4 h after immunization (Fig. 2s) suggesting that follicular B cells deliver antigen to follicles and that this Ag is subsequently transferred to CD8α^−^ cDCs and presented to CD4^+^ T cells. Transfer and endocytosis of IgE-Ag by the CD8α^−^ cDCs cannot be dependent on CD23 since these cells do not express CD23 (Fig. 4). The most straightforward explanation for the delivery process, and the one we favour, is that the dendritic cells sample the environment ‘as usual’^45^ and, since immunization with IgE-OVA complexes increases the local concentrations of Ag tremendously (Fig. 2b,f,c,g), more Ag is available for uptake and presentation. A mutually not exclusive possibility is that exosomes are involved. B cells can process IgE-Ag complexes into exosomes in a CD23-dependent manner and dendritic cells, co-cultured with such exosomes and transferred to mice, induce proliferation of specific T cells^46^. Dendritic cells express several different FcγRs, among which FcγRII, FcγRIII, and FcγIV can bind IgE^47^,^48^,^49^. Therefore, IgE-Ag complexes could be transferred from CD23 on B cells to FcγRs on CD8α^−^ cDCs and trigger endocytosis and presentation of peptides on MHC-II. However, this possibility is unlikely since IgE-mediated enhancement operates well in mice lacking functional FcγRIII, FcγIV, and FcεRI (FcRγ^−/−^) as well as in mice lacking FcγRII^50^.

The data presented here and elsewhere suggest the following scenario for IgE-mediated enhancement of immune responses. Ag enters the blood, forms a complex with IgE and binds to CD23^+^ B cells^6^,^37^ (or, hypothetically, a pathogen expressing the appropriate lectins binds directly to CD23^+^ B cells). These recirculating B cells have access to splenic follicles and substantial amounts of Ag is found on follicular B cells in the spleen, and is detected in splenic follicles, 0.5–4 h after immunization^6^ (Fig. 2). CD11c^+^ cells^7^, more precisely CD8α^−^ cDCs (Fig. 3), acquire Ag in this environment and present it to specific CD4^+^ T cells^5^,^7^ (Figs 1b,c and 3) which efficiently help cognate B cells, resulting in the enhanced formation of germinal centres^6^ and in the enhanced primary and secondary antibody responses^2^,^3^,^4^,^5^,^29^,^30^.

A relevant question is what the biological significance of the described sequence of events could be. Although serum concentrations of IgE are generally low, levels are elevated both in parasite infections and allergies and IgE can be produced after viral infections^51^. Moreover, local IgE concentrations may be much higher than serum concentrations. An interesting possibility is that IgE itself may not even be required for binding of an Ag to CD23: unlike other Fc-receptors, CD23 belongs to the C-type lectin family and it has been speculated that the ability of CD23 to bind IgE is just a fortuitous function, and that more common ligands are carbohydrates. Other members of the C-type lectin family act as pattern recognition receptors^52^ suggesting that also CD23 may bind directly to microbes. Since recirculating B cells expressing high levels of CD23 have access to splenic follicles, this would hypothetically constitute a rapid transportation route for blood-borne pathogens binding directly to CD23, analogous to the route for blood-borne IgE-Ag complexes demonstrated herein.

Unlike murine DCs, human DCs express both CD23 and FcεRI and therefore handling of IgE-Ag immune complexes by DCs may differ between these species. Recent studies in humanized mice, carrying a human FcεRIα-transgene, indicate that targeting Ag to DCs results in tolerization of certain T cell populations^53^,^54^. Notably, a fusion protein consisting of human IgE Fc parts (Cε2–Cε4) fused to an OVA peptide, and not native IgE-Ag complexes, was used in this study^53^. Clearly, both human and murine B cells can bind IgE-Ag via CD23 and present Ag to CD4^+^ T cells *in vitro*^9^,^10^,^11^,^12^,^13^, but whether human B cells also transport IgE-Ag complexes is not known.

## Methods

### Mice

BALB/c mice were obtained from Bommice (Ry, Denmark). DO11.10, carrying a transgenic T cell receptor (TCR) recognizing an OVA peptide bound to I-A^d^ class II molecules^55^, and CD23^−/−^ 4 mice were kind gifts from Dr Westerberg (Karolinska Institute, Stockholm, Sweden) and Dr Kishimoto (Osaka Medical Center, Osaka, Japan) respectively. CD23^−/−^ mice were backcrossed to BALB/c for 11 generations and all mice were bred and maintained at The National Veterinary Institute (Uppsala, Sweden). All animal experiments were approved by Uppsala Animal Research Ethics Committee (permit number C25/13). All experiments were carried out in accordance with the approved guidelines. Mice used within each experiment were matched for sex and age.

### Preparation of IgE anti-OVA mAb and culture medium

Mouse IgE anti-OVA mAb used for immunization was purified from supernatant of the TOε hybridoma (a kind gift from Dr Bryce, Northwestern University, Illinois)^56^ by affinity chromatography over OVA-coupled CNBr-activated Sepharose 4B (GE Healthcare Life Sciences, Uppsala, Sweden). Complete culture medium for the hybridoma consisted of RPMI-1640 supplemented with 10% heat-inactivated fetal calf serum, 10 mM HEPES, 100 U/ml penicillin, 100 μg/ml streptomycin, 50 μM 2-mercaptoethanol, 1 mM sodium pyruvate and 2 mM L-glutamine (all from Sigma-Aldrich, St Louis, MO). The same complete culture medium was used for *ex vivo* T cell proliferation assays.

### Immunization

Mice were immunized i.v. with OVA (Grade V; Sigma-Aldrich) or OVA-Alexa 647 (Life technologies, Carlsbad, CA) alone, or with IgE anti-OVA pre-mixed with OVA or OVA-Alexa 647 in 200 μl PBS. The doses used are indicated in each figure.

### Flow cytometry

Single cell suspensions were prepared as described^7^ and blocked with anti-CD16/CD32 mAb (clone 2.4G2; BD Biosciences, San Diego, CA) before staining with mAb or dye. PE-labeled anti-CD4 (clone GK1.5), PE- or PE-Cyanine7-labeled anti-CD11c (clone N418), PE-Cyanine7-labeled anti-PDCA-1/BST2/CD317 (clone eBio927), Alexa Fluor 700-labeled anti-CD11b (clone M1/70), PE-Cyanine5-labeled anti-CD45R/B220 (clone RA3-6B2) and PE-labeled anti-CD23 (clone B3B4) mAb and PE-labeled streptavidin were from eBioscience (San Diego, CA). PE- or FITC-labeled anti-MHC-II (I-A^d^; clone AMS-32.1), Alexa Fluor 647-labeled anti-DO11.10 TCR (clone KJ1-26) and biotinylated anti-CD86 (clone GL1) mAb and Fixable Viability Stain 450 (FVS450) were purchased from BD Biosciences. Alexa Fluor 488-labeled anti-DCIR2 (dendritic cell inhibitory receptor 2; clone 33D1) and Brilliant Violet 421-labeled anti-CD8α (clone 53-6.7) mAb were from Biolegend (San Diego, CA). FITC-labeled anti-DO11.10 TCR (clone KJ1-26) mAb was obtained from Life technologies. Events were acquired on a LSR II cytometer (BD Biosciences) and data were analysed by FlowJo software (Tree Star, Ashland, OR). Cells were gated for singlets according to their forward- and side-scatter properties prior to analysis as shown in Supplementary Fig. S2a.

### Adoptive transfer and *in vivo* CD4^+^ T cell proliferation assay

Mice were transferred i.v. with 3 × 10^6^ CD4^+^ T cells, isolated using anti-CD4-conjugated magnetic beads (Miltenyi Biotec, Bergisch Gladbach, Germany) and MACS, or 15 × 10^6^ total cells from DO11.10 mouse spleens in 200 μl PBS and immunized the next day. Three days after immunization, spleens were harvested and analysed by flow cytometry for OVA-specific CD4^+^ T cells after staining with the clonotypic KJ1-26 mAb, recognizing the OVA-specific TCR on DO11.10 T cells^7^.

### *Ex vivo* CD4^+^ T cell proliferation assay

Four hours after immunization, spleens from BALB/c mice were removed and splenocytes were stained for fluorescence-activated cell sorting. CD8α^−^ cDCs (PDCA-1^−^CD11c^high^MHC-II^high^CD11b^+^CD8α^−^), CD8α^+^ cDCs (PDCA-1^−^CD11c^high^MHC-II^high^CD11b^−^CD8α^+^), pDCs (PDCA-1^+^CD11c^int^MHC-II^int^) or CD8α^−^DCIR2^+^ cDCs (CD11c^high^MHC-II^high^CD11b^+^CD8α^−^DCIR2^+^) were sorted with a FACSAriaIII cell sorter (BD Biosciences). CD4^+^ T cells were isolated from splenocytes of DO11.10 mice using anti-CD4-conjugated magnetic beads (Miltenyi Biotec) and MACS. Isolated CD4^+^ T cells were labeled with 5 μM CFSE (Life technologies) as described before^5^. Cells were washed 3 times in complete culture medium before use. Each subset of the sorted dendritic cells (5000 or 1200 per well) was then cultured together with 5000 CFSE-labeled CD4^+^ T cells in 96-well U-bottom plates (Sarstedt, Nümbrecht, Germany), resulting in an APC:T ratio of 1:1 or approximately 1:4. CD4^+^ T cells cultured alone were used as negative controls and all CD4^+^ T cells in each experiment were from the same preparation. Cells were incubated for 3 days at 37 °C, and then stained and analysed by flow cytometry for OVA-specific CD4^+^ T cell proliferation.

### Confocal microscopy

Spleen samples were embedded in optimal cutting temperature embedding media (VWR International, Radnor, PA) and snap-frozen in liquid nitrogen. Eight-micrometer sections were prepared with CryoStar NX70 Cryostat (Thermo Scientific, Waltham, MA), air-dried and stored at −80 °C. Sections were fixed in 4% paraformaldehyde (Merck, Darmstadt, Germany) for 15 min before blocking with 5% horse serum (Sigma-Aldrich) for 30 min. Pacific Blue-labeled anti-CD45R/B220 (clone RA3-6B2; BD Biosciences) for staining B cells and FITC- or Alexa Fluor 647-labeled anti-CD169 (clone MOMA-1; AbD Serotec, Oxford, UK) for staining metallophilic macrophages in the marginal zone were used. OVA-specific T cells were stained with biotinylated anti-DO11.10 TCR mAb (clone KJ1-26; eBioscience) followed by streptavidin-conjugated PE (eBioscience). DCIR2^+^ cDCs were stained first with biotinylated rat anti-DCIR2 mAb (clone 33D1; Biolegend) and then with secondary biotinylated anti-rat IgG (eBioscience) followed by streptavidin-conjugated PE (eBioscience). The sections were mounted with Fluoromount G (Southern Biotech, Birmingham, AL) after staining and washing, and analysed with an LSM 700 confocal microscope (Carl Zeiss, Thornwood, NY). Images taken were processed and analysed with ImageJ software (National Institutes of Health, Bethesda, MD).

### ELISA

Mice were bled from the tail artery at indicated time points and sera were prepared and stored at −20 °C. IgG anti-OVA ELISA was performed as described^37^. Construction of standard curves and calculations of the concentration of IgG anti-OVA were performed with SOFTmax software (Molecular Devices, Sunnyvale, CA).

### Statistical analysis

Statistical differences between groups were determined by unpaired Student’s *t*-test (two-tailed) with Prism 5.0d (GraphPad Software, La Jolla, CA). P-values < 0.05 were considered significant.

## Additional Information

**How to cite this article**: Ding, Z. *et al*. IgE-mediated enhancement of CD4^+^ T cell responses requires antigen presentation by CD8a^−^ conventional dendritic cells. *Sci. Rep.* **6**, 28290; doi: 10.1038/srep28290 (2016).

## Supplementary Material

Supplementary Information
