# Supplementary material for: IgE-mediated enhancement of CD4+ T cell responses requires antigen presentation by CD8α− conventional dendritic cells
Source: Sci Rep. 2016 Jun 16;6:28290. doi: 10.1038/srep28290 (PMC4910288; doi:10.1038/srep28290)
Supplement: Supplementary Information [file srep28290-s1.pdf]

IgE-mediated enhancement of CD4$^+$ T cell responses requires antigen presentation by CD8α$^-$ conventional dendritic cells

Zhoujie Ding[1], Joakim S. Dahlin[1], Hui Xu[1] and Birgitta Heyman[1,*]

[1] Department of Medical Biochemistry and Microbiology, Uppsala University, Uppsala, Sweden

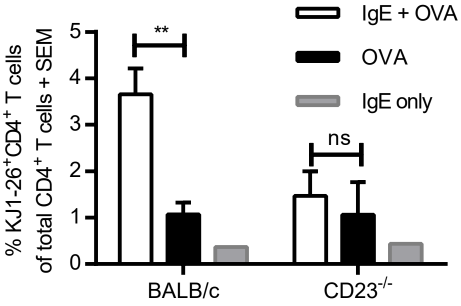

Supplementary Fig. S1. The enhancement of CD4$^+$ T cell responses by IgE is abolished in CD23$^{-/-}$ mice. BALB/c and CD23$^{-/-}$ mice were adoptively transferred with 3×10^6 CD4$^+$ T cells from DO11.10 mouse spleens. The next day, mice were immunized with 50 μg IgE anti-OVA pre-mixed with 20 μg OVA (n=3) or 20 μg OVA alone (n=3). One mouse immunized with 50 μg IgE anti-OVA alone was used as negative control (n=1). Spleens were harvested 3 days after immunization and analysed for proliferation of OVA-specific CD4$^+$ T cells by flow cytometry. The gating strategy is shown in Supplementary Fig. S3. Percentages of KJ1-26$^+$CD4$^+$ T cells among total CD4$^+$ T cells of each group were quantified. Data are from one experiment and are shown as mean + SEM. Significance was determined between the groups immunized with IgE-OVA complexes and OVA alone by Student's *t*-test. **, $p < 0.01$; no significance (ns), $p > 0.05$.

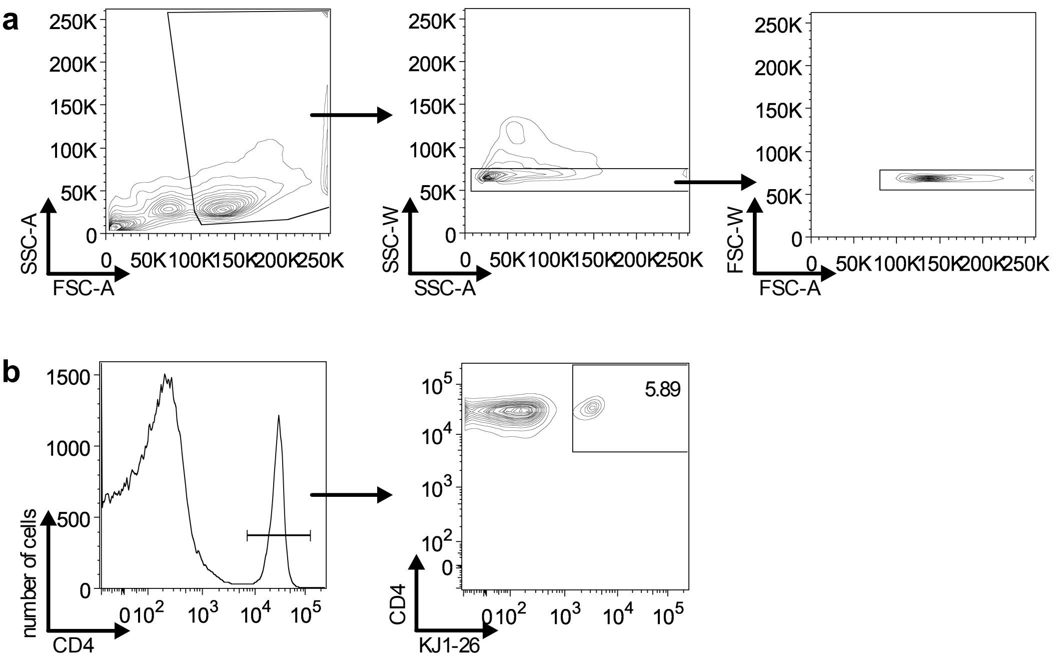

Supplementary Fig. S2. Gating strategy of OVA-specific CD4$^+$ T cells. (a) Cells were gated for singlets according to their forward- and side-scatter properties prior to analysis. (b) CD4$^+$ T cells were gated compared to internal negative population (left panel). OVA-specific CD4$^+$ T cells were then gated as KJ1-26$^+$CD4$^+$ cells (right panel). Number indicates the percentage of KJ1-26$^+$CD4$^+$ T cells among total CD4$^+$ T cells.

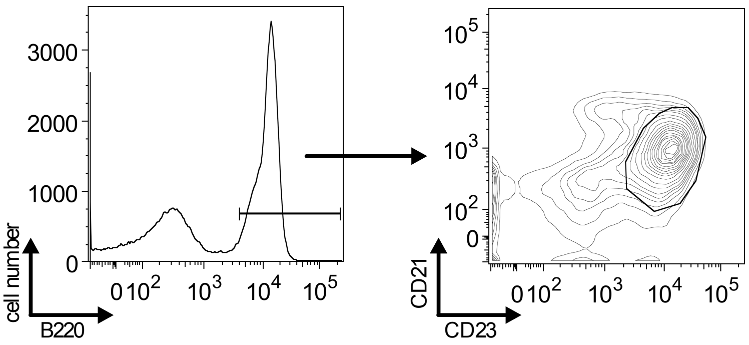

Supplementary Fig. S3. Gating of follicular B cells. B220$^+$ cells were gated compared to internal negative population (left panel). Follicular B cells were then gated as CD21$^+$CD23^{high} cells among the B220$^+$ cells (right panel).

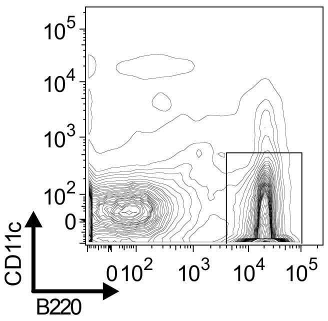

Supplementary Fig. S4. Gating of B220^{+}CD11c^{-} B cells.
